# Safety and efficacy of fiducial marker implantation for robotic stereotactic body radiation therapy with fiducial tracking

**DOI:** 10.1186/s13014-019-1373-2

**Published:** 2019-09-13

**Authors:** Nathaniel Scher, Marc Bollet, Gauthier Bouilhol, Remi Tannouri, Imane Khemiri, Aurelie Vouillaume, Noura Sellami, Rie Von Eyben, Jean-Michel Vannetzel, Ilan Darmon, Luc Rotenberg, Hanah Lamallem, Olivier Bauduceau, Denis Foster, Alain Toledano

**Affiliations:** 1 Hartmann Radiotherapy Institute, Levallois-Perret, 4 rue Kleber, Levallois-Perret, France; 2 Rafael Institute, Center for Predictive Medicine, 3 boulevard Bineau, Levallois-Perret, France; 3 Department of Statistical Analysis, Radiation Oncology, Stanford University School of Medicine, 875 Blake Wilbur Drive, California, USA

**Keywords:** Stereotactic body radiation therapy, Image guided radiation therapy, Fiducial marker

## Abstract

**Purpose:**

The purpose of this study was to assess the feasibility, efficacy and toxicity of fiducial marker implantation and tracking in CyberKnife® stereotactic radiation therapy (SBRT) applied to extracranial locations.

**Materials and method:**

This is a retrospective, single-centre, observational study to collect the data of all patients treated by stereotactic radiation therapy with fiducial marker tracking at extracranial locations, conducted between June 2014 and November 2017. Information regarding the implantation procedure, the types of toxicity related to marker implantation and the number of markers implanted/tracked during treatment were collected. Complication rates were evaluated using the CTCAE v4 [Common Terminology Criteria for Adverse Events] scale. The technical success rate was based on the ability to optimally track the tumor throughout all treatment fractions.

**Results:**

Out of 2505 patients treated by stereotactic radiation therapy, 25% received treatment with fiducial marker tracking. The total number of implantation procedures was 616 and 1543 fiducial markers were implanted. The implantation-related complication rate was 3%, with 16 Grade 1 events and 4 Grade 2 events. The number of treated patients and the number of implanted markers has gradually increased since the technique was first implemented. The median treatment time was 27 min (range 10–76). 1295 fiducials were effectively tracked throughout all treatment fractions, corresponding to a technical success rate of 84%. The difference between the number of fiducials implanted and those tracked during treatment decreased significantly as the site’s experience increased.

**Conclusion:**

Fiducial marker implantation and tracking is feasible, well-tolerated, and technically effective technique in SBRT for extracranial tumors.

## Background

Extracranial radiation therapy under stereotactic conditions (Stereotactic Body Radiation Therapy, or SBRT) is defined as a high-precision treatment method that delivers a high dose of radiation to a target volume with a steep dose gradient, ensuring that surrounding critical organs are spared. Organ movement and deformation between and during treatment fractions must be characterized, verified, and taken into account during treatment planning. This challenge has led to the development of several methods to monitor the position of the target and normal tissue avoidance structures. Image-guided radiotherapy (RT) is the use of various imaging modalities before or during radiotherapy treatments to align and verify the anatomical agreement between simulation anatomy, treatment of RT and the patient at the time of treatment. The image guided in SBRT (IG-SBRT) is necessary to ensure accurate target coverage despite the necessary reductions in target volume margins expected to reduce the dose to the organs at risk.

Imaging techniques may be divided into radiation-based and non-radiation-based systems. Non-radiation-based systems include electromagnetic tracking, ultrasound and MRI systems integrated into treatment machine. Radiation-based systems include static as well as real time tracking, using either kV, MV, or hybrid methods. Registration of tumor targets and normal tissue avoidance structures to the treatment delivery machine is possible via motion control devices. There are three categories of motion control techniques: the gating, the damping and the tracking. The gating consist to follow the respiratory cycle using a surrogate in order to trigger the beam only in a specific time. The damping device such as abdominal compression or breath hold maneuvers can restrain the motion. Tracking device make possible to move the radiation beam to follow the moving target.

The CyberKnife® allows for frameless SBRT of extracranial locations with real-time target and motion tracking using Kv imaging methods. Two target tracking methods are currently available with this device: the fiducial tracking that requires the use of fiducial markers which are radio-opaque markers implanted around or within the tumor, and the Xsight® Lung Tracking System that is fiducial-free. Both tumor tracking methods can be combined with the Synchrony® Respiratory Tracking System, which synchronizes the beam delivery with the motion of the target due to respiration. Trackable fiducials, whose position can be measured rapidly, accurately, and objectively by a fiducial tracking system contribute for accurate patient positioning and targeting because they can provide the accuracy of fiducial-based IGRT while remaining as fast, easy, and objective as skin mark alignment. However, SBRT with fiducial tracking do not require and is not defined by one device. Each fiducial marker must remain in a fixed position in relation to the tumor and to the other markers. This ensures tracking of target translation movements (if at least one fiducial is tracked) and rotational movements (if at least three fiducials are tracked). Because the geometrical relationship between the fiducial marker and the target volume represents the greatest uncertainty in the CyberKnife® tracking system, optimal placement of the markers is essential [1]. Sub-optimal final positioning of one or more markers is often inevitable. To resolve this, additional markers are frequently implanted as back-ups. Despite growing interest for SBRT treatment of extracranial tumors, few institutions have accurately reported any practical experience with fiducial implantation and tracking in SBRT.

The purpose of this study was to assess the feasibility, efficacy and toxicity of fiducial marker implantation and tracking in CyberKnife® stereotactic radiation therapy applied to extracranial locations.

## Methods

This is a retrospective, single-centre, observational study to collect the data of all patients treated by stereotactic radiation therapy with fiducial marker tracking at extracranial locations, conducted between June 2014 and November 2017 at the Hartmann Radiotherapy Institute. Patients requiring fiducial implantation were selected at the SBRT dedicated meetings. Information about the physician who performed the procedure, the implantation path, the success rates/rates of implantation-related complications, and the number of markers implanted/tracked during treatments were collected for all patients in whom fiducials were implanted. The success rate is defined by the number of fiducial markers implanted in relation to the number of markers actually tracked throughout the treatment. Thus, for each patient, the number of fiducials that the CyberKnife® were not able to track has been recorded. This study was approved by our Institutional Clinical Research Committee.

### Implantation procedure

Eight different radiologists performed the implantation procedures. The implantation procedure and the type of fiducials implanted evolved over time, according to the experience acquired by the radiologist. The exclusion criteria were the same as for a biopsy [2].

All procedures were image-guided and performed on patients under short-duration local or general anaesthesia. Images were taken before, at regular intervals during, and following the procedure to check for fiducial positioning and the absence of immediate complications.

The fiducials were implanted close to the tumor, observing as closely as possible the fiducial implantation recommendations [3]. Each patient underwent a planning CT scan, required for treatment planning, using an Optima CT580 scanner (General Electric Company, Waukesha, WI) with a 4D option and 1.25 mm cross-section thickness. All treatments were performed using a CyberKnife® M6 FIM System (Accuray, Sunnyvale, CA) in combination with a robotic treatment table (RoboCouch), with fixed collimators, an IRIS collimator, an MLC collimator, and 6DSkull, Fiducial, Synchrony, Lung Optimized Treatment, Xsight Lung, XSight Spine and XSight Spine Prone tracking modes. The target is tracked throughout the treatment and delivery is automatically altered to compensate for any motion. The image guidance system calculates the required offsets based on the patient’s current position. During the delivery of the treatment the system automatically corrects the linac position for any calculated offsets within a specified tolerance. The offset tolerance for intra-fraction robotic corrections are 10 mm for X,Y and Z and 1.5° for pitch roll and Yaw; except for the prostate which the tolerance are 1.5°, 2° and 3° respectively.

## Results

**Fig. 1:**
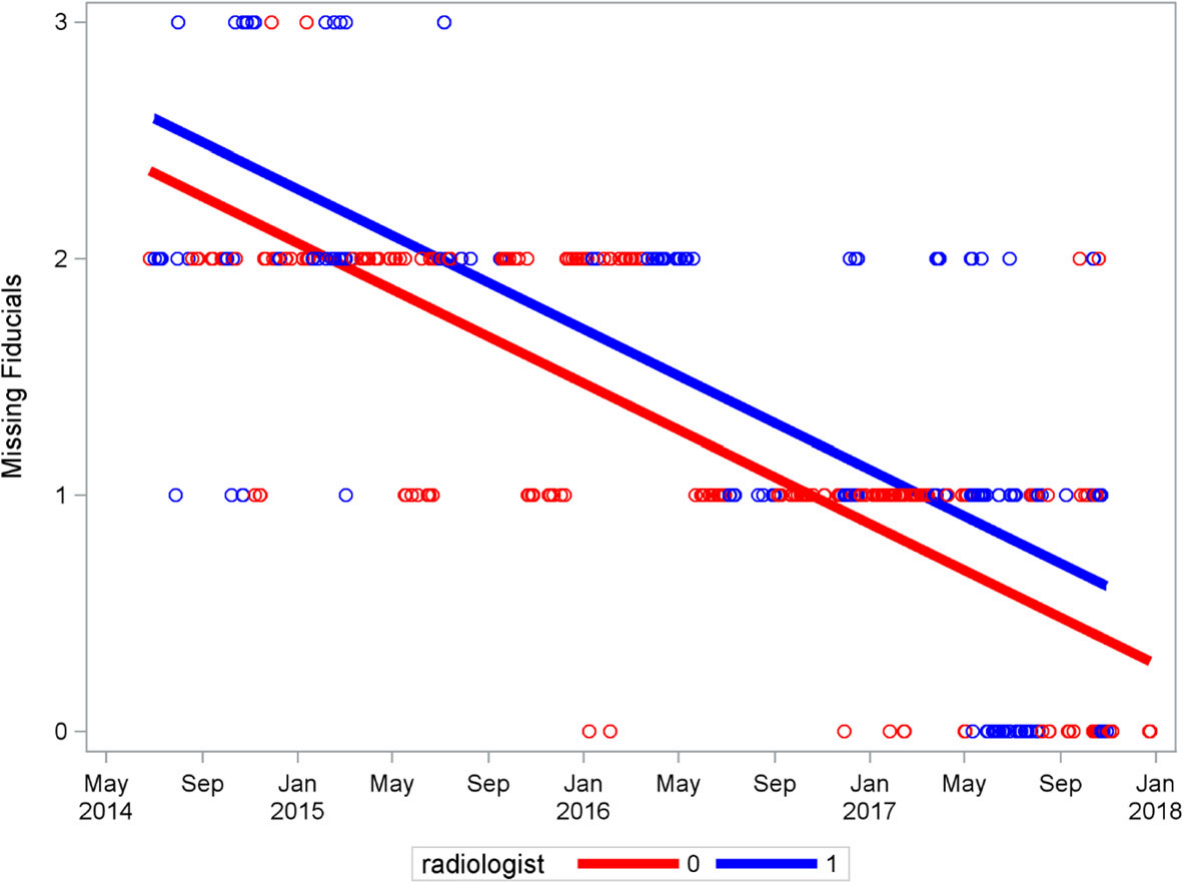
analysis of covariance for missing fiducials over time. Radiologist 0: main radiologist. Radiologist 1: the seven other radiologists pooled together. There is a significant decrease in the number of missing fiducials over time, *p* < 0.0001. The main radiologist had significantly fewer missing fiducials than the other radiologists, *p* < 0.0001

Out of 2505 patients were treated by stereotactic radiation therapy and 25% of them (616 patients) have received IG-SBRT for extracranial locations with fiducial marker tracking. Thus, 1543 fiducial markers were implanted and 2757 sessions with fiducial tracking were performed. The median patient age at the time of treatment was 71 years (range [33–94]). The median time intervals between fiducial implantation, the simulation scan and the first treatment session were, respectively, 11 days (range [5–41]) and 15 days (range [2–36]). The median total prescribed dose was 36 Gy (mean 39 Gy, SD 10.2, range [15–60]), the median fractional dose was 10 Gy (mean 11, SD 1.6, range [6–20]) and the median number of fraction was 5 (mean 4.7, SD 1.9, range [3–6]). The median duration for one treatment was 29 mn (mean 27, SD 1.5, range [10–76]). The rate of implantation-related complications was measured at 3%, with 16 Grade 1 events, 4 Grade 2 and a single grade 3 event. The number of patients treated and the number of implanted markers increased since the technique was first implemented, with close to 350 patients treated with fiducial tracking over the year 2017. 468 procedures, i.e. 76% of all procedures, were performed by the same radiologist. 1295 fiducials were actually tracked throughout all treatment fractions, representing 84% of the implanted fiducials. Moreover, there is a significant decrease in the number of missing fiducials over time, *p* < 0.0001, indicating a learning curve by the radiologists. The main radiologist had significantly fewer missing fiducials than the other radiologists, *p* < 0.0001. On average this radiologist had 0.23 fewer fiducials missing and this difference persisted across all the measured dates (Fig. 1). The toxicities related to the implantation are not statistically different according to the radiologist, *p* = 0.15.

All results are summarised in Tables 1 and 2.

**Table 1:** Outcomes of fiducial markers implementation and tracking for robotic extracranial SBRT

Outcomes	*n*	Grade 1, *n*	Grade 2, *n*	Grade 3 or more, *n*	Technical success, %
Total procedures	616	14	6	1	84%
Lung	89	8	2	0	100%
Liver	151	0	2	0	75%
Prostate	150	5	0	0	91%
Nodes	85	0	1	0	100%
Pancreas	18	0	1	0	85%
Bones	57	0	0	0	100%
Adrenals	23	0	0	0	100%

**Table 2:** Outcomes about fiducial markers implanted and tracked according to localization treated

Localization and number of patient (*n*)	Fiducial markers implanted Median,	Fiducial markers tracked Median,
	SD,	SD,
	range	range
Lung *n* = 89	1	1
	0.3	0.3
	[1, 2]	[1, 2]
Prostate *n* = 150	4	3
	0.8	0.5
	[2–5]	[2–4]
Liver *n* = 151	4	3
	1.3	1
	[1–6]	[1–5]
Nodes *n* = 85	1	1
	0.6	0.6
	[1–3]	[1–3]
Pancreas *n* = 18	3	3
	1.1	0.5
	[1–5]	[1–3]
Bones *n* = 57	1	1
	0	0
	1	1
Adrenal *n* = 23	2	2
	0.9	0.9
	[1–4]	[1–4]

## Lungs

### Patients and methods

Out of 450 patients treated by lung SBRT for primary or secondary tumors, 89 received fiducial implantations. 15 patients had previously received lung SBRT without fiducial implantation. The fiducial implantation procedure consisted in implanting a single ‘Concerto’ or ‘Gold Anchor’-type seed through guided needle puncture. Fiducial implantation in the lungs was generally performed percutaneously via the trans-thoracic route, or through endovascular access if the patient had severe respiratory co-morbidities (single lung, major emphysema).

### Results

One hundred two fiducials were implanted and 89 patients were treated. The procedure was performed by the same radiologist in 69 patients, i.e. in 78% of the procedures. The trans-thoracic implantation route was used in 90%, and the endo-vascular route was used in 10% of the implanted patients. All seeds were correctly positioned in the close vicinity of the tumor. In two of the 89 patients, a second seed had to be implanted within the same operative step due to immediate migration of the first seed. The median number of implanted and tracked fiducials per patient was, respectively, 1, mean 1.1, SD 0.32, range [1, 2].

The implantation procedures were complicated by eight Grade 1 events: seven cases of pneumothorax occurring immediately after the puncture, not requiring a drain, and one episode of minimal haemoptysis. The implantation procedures were complicated by two Grade 2 events: two cases of pneumothorax occurring immediately after the puncture and requiring a drain. No cases of lung bleeding were reported. No events of Grade 3 or higher were reported. No patients required surgery or intensive care hospitalisation. No cases of secondary migration were observed on the tracking scan. The median dose was 60 Gy (range [36–60]) and the median number of fraction was 3 (range [3–6]). All implanted fiducials were used to reliably determine the lesion location, ensuring effective tracking and continuous radiation of the lesion throughout the treatment. The technical success rate was, therefore, 100%.

## Prostate

### Patients and methods

All patients undergoing SBRT treatment in the prostate underwent fiducial implantation. The procedure consisted in placing non-joined seeds *in the years 2014 and 2015*, and later using joined *Fleximark*-type seeds. The fiducials were implanted transperineally in the prostate in the transverse and sagittal planes under endorectal ultrasound guidance.

### Results

One hundred fifty patients received SBRT treatment with fiducial implantation and tracking for prostatic tumor. The median dose was 36 Gy (range [36–36.25]) and the median number of fraction was 6 (range [5, 6]). In total, 544 seeds were implanted, i.e. a median number of 4 seeds per patient, mean 3.6, SD 0.84, range [2–5]. 88 procedures were performed by the same radiologist (58%). The tolerance rate was excellent. The implantations were complicated by seven Grade 1 events: 4 transient haematuria events and 3 perineal pain events. In addition, one fiducial had migrated out of the prostate and into the perineum immediately after implantation.

Four hundred seventy fiducials were tracked, i.e. 86% of the implanted fiducials. The total median number of fiducials tracked per patient was 3, mean 3.1, SD 0.57, range [2–4]. For 14 patients, two of the four implanted fiducials could not be selected at the planning stage, and therefore during tracking, as they were placed too close together or in a co-linear pattern. For all other patients (136 patients), at least three fiducials were tracked throughout the treatment. All fiducials selected at the planning stage were actually tracked during the treatment sessions. No fiducial migration was observed between the tracking scan and the last treatment fraction. The technical success rate was measured at 91%.

## Liver

### Patients and methods

Fiducial implantation was performed under arterial CBCT guidance, percutaneously and/or endovascularly, depending on tumor location and patient co-morbidities. The endovascular route was preferred, particularly for centrally located tumors. The advantage of the endovascular route is that is provides a better view of the tumor volume shown by the contrast medium injection. The radiologist selected the level of scan sections and the puncture site. Under radiological guidance, the radiologist performed 2 needle punctures, dropping one to two joined seeds via the needle. The markers were placed perpendicularly according to a cube-shaped pattern centred around the tumor. The edges of the cube had to be in two different spatial planes.

### Results

One hundred fifty-one patients received SBRT treatment with fiducial implantation and tracking for primary or secondary tumoral lesions of the liver. The median dose was 45 Gy (range [40–45]) and the median number of fraction was 3 (range [3–5]). Six hundred seventeen fiducials were implanted, with a median number of 4 implanted fiducials per patient (mean 4, SD 1.26, range [1–6]). One hundred ten procedures (73%) were performed by the same radiologist. The tolerance rate of the implantation procedure was excellent. Fiducial implantation was complicated by two Grade 2 events, i.e. a sub-capsular haematoma in 2 patients. In addition, three cases of secondary migration inside the liver were noted: one occurring during the implantation procedure and two secondary migrations observed on the dosimetry scan. Finally**,** 461 fiducials were tracked, i.e. 74% of the implanted markers, with a median number of 3 tracked fiducials per patient (mean 3, SD 1, range [1–5]). The technical success rate was, therefore, 75%.

### Other locations

Outcomes about fiducial markers implanted and tracked into lymph nodes, pancreas, bones and adrenal glands are summarized in Tables 1 and 2.

## Discussion

Implantation of fiducial markers for SBRT treatment of extracranial targets is a feasible, well-tolerated and effective technique, as summarised in the Table 3.

**Table 3:** Summary of the outcomes about fiducial markers implantation and tracking concerning the three main tumor localization: lung, prostate and liver

	Patients treated with SBRT (*n*)	Patients treated with fiducial tracking (*n*, %)	Fiducial markers implanted (*n*)	Fiducial markers tracked (*n*)	Technical sucess rate (%)	⩾ grade 2 toxicities (%)
Lung	450	89 (20%)	102	102	100%	2%
Prostate	160	150 (94%)	544	470	91%	0%
Liver	165	151 (92%)	617	461	75%	1%

To our knowledge, the studied cohort of 616 patients who received SBRT treatment with fiducial tracking is the largest reported in the literature. Such numbers provide grounds for a reliable and comprehensive evaluation of fiducial implantation and tracking practices, since all extracranial locations were analysed. The uniqueness of this study is that it objectively evaluates the number of fiducials actually tracked for each patient during treatment, and therefore provides data not only on the toxicity profile of the implantation procedure, but also on the efficacy of fiducial tracking and the technical success of SBRT treatment. Thus, we propose that four fiducials markers need to be implant in order to track three markers concerning prostate and liver target. We also suggest that joined markers be used whenever technically possible to reduce the number of needles and therefore puncture-related complications.

Because fiducial implantation is an invasive technique, it must not only ensure optimal tumor tracking, but also be acceptable for patients and involve minimal complications. It has been shown that fiducial tracking allow for high precision tumor localization and real time target tracking without the need to restrain the patient’s breathing. In this study, implantation and tracking of fiducial marker is safe because the reported rate of grade 2 or more related toxicities is 1% with six grade 2 and one grade 3 events. The size of the dataset is relevant to have a representative sample of toxicities in this setting. The safety seems good enough to justify using this invasive technique regarding the other options. However, fiducial placement has some drawbacks. It is an invasive procedure and not applicable for all patients and thus limits the application range of fiducial tracking SBRT. Otherwise, fiducial markers alter the image quality of the planning CT and make sometimes the tumor delineation more difficult.

Many studies define the technical success of an implantation procedure as the fact of placing at least one fiducial in the vicinity of the target [4, 5]; other studies refer to the number of implanted fiducials [6]. The power of our study lies in the fact that it practically evaluates technical success based on the number of fiducials tracked throughout the treatment, since in order to ensure optimal target tracking, some tumor locations (prostate, liver, pancreas) require tracking of rotational movements in addition to translation movements throughout all the treatment fractions. To achieve this, at least three markers need to be tracked within each treatment fraction. To this day, no published study has reported the number of fiducials tracked during SBRT treatment in different locations. In addition, it is important to look at the proportion of fiducials that are actually tracked during treatment compared to the number of implanted fiducials and to take into account this difference in order to restrict the number of implantation invasive procedure and to conduct an assessment of professional practices. Several reasons may explain the fact that an implanted marker may not always be detected and tracked: 1) Marker migration; 2) Co-linear markers; 3) Deformation of an organ causing a change in the geometrical relationship among markers. All these scenarios can cause the CyberKnife® imaging system to no longer recognise some markers, and therefore to be unable to track them.

This is due to the fact that the CyberKnife® fiducial tracking method has a weakness:

It considers the geometrical relationship among fiducials as rigid, and the geometrical relationship between the fiducials and the target as invariant. Thus, it does not take into consideration the possibility of anatomical deformations which can change the geometrical relationships among the fiducials themselves, or between the fiducials and the target.

This study also demonstrates the learning curve of the team translating in an improved efficacy of fiducial marker tracking as experience is gained. There has been a decrease in the difference between the number of implanted fiducials and the number actually tracked during treatment in the 2 years since this technique was implemented in our department. There is a significant decrease in the number of missing fiducials over time. This learning curve can be explained by several reasons. First, increasing the number of implantation procedure make it technically more efficient. Second, regular team meetings provide feedback and solutions to radiologists to improve placement. Continuous interactions between the members of the treatment team – radiographers, medical physicists, radiation oncologists, radiologists – help to identify the difficulties due to fiducial implantation and tracking, and to resolve them depending on treatment constraints Third, the implantation equipment has improved and adapted to the demand over time (pre-loaded needle, set of linked markers). Thus, radiologists gather technical expertise and achieve to be more efficient according recommendations over time as the proportion of tracked vs. implanted fiducials becomes greater.

The toxicities related to the implantation are not statistically different according to the radiologist (p = 0.15). However, the rates of toxicity decrease overtime with 9, 7 and then 5 events over the three and half years. The event rates are too low to show a statistically significant difference. This study demonstrates the learning curve of the radiologist translating in an improved efficacy of fiducial marker tracking and probably in toxicities related to implantation as experience is gained. Thus, we suggest a minimum of 100 implantations procedure per year to ensure a high quality of fiducial tracking SBRT program. We recommend multi-disciplinary collaboration for implementation and application of fiducial tracking SBRT. Each patient’s proposed course of SBRT treatment should be discussed and reviewed by the multi-disciplinary stereotactic team. We think useful to develop processes for initial and ongoing training, to write departmental protocols, to structure follow up for assessment of the toxicity and efficacy of the procedure in order to evaluate and improve continuously clinical practices.

A medical-economic evaluation covering the management of implantation complications, the number of fiducials to be implanted, and the number of postponed treatment sessions should, logically, demonstrate that ensuring a lower level of toxicity and higher efficacy in fiducial implantation and tracking will also help reduce per-treatment costs as the number of patients under treatment gradually increases [7].

This study has certain limits. Firstly, it is a retrospective and single-centre study, which means the level of data evidence reported is low. However, the goal of the study was to report a preliminary experience based on a large patient population, while remaining strict on data collection quality and toxicity ratings, in order to provide reliable and comprehensive results. In addition, no long-term treatment results are reported in this cohort. This type of results, however, regarding SBRT treatment for different tumor locations is already well known, and the purpose of this work was to focus on the feasibility and toxicity of fiducial implantation and the efficacy of fiducial tracking during treatment. Lastly, successfully tracked fiducials do not guarantee good localization. Indeed, the fiducials were implanted close to the tumor, and he distance between the fiducials and the target was not recorded. It would be interesting to collect data related to this parameter in order to report on implantation quality and target tracking reliability.

### Lung

The rate grade 2 or more implantation related complications was very low (2%) in this report. Trans-thoracic fiducial implantation is responsible for a pneumothorax rate that varies across the different studies from 10 to 23% [8–11]. In a population that is generally frail, ageing, often with strong vascular, and particularly pulmonary, histories (such as chronic obstructive pulmonary disease), the risk of pneumothorax should be taken into consideration. Using the endo-vascular route as an alternative can reduce complications. No complications during marker implantation via the endo-vascular route were collected in our study, and the literature reported complications such as pulmonary infarction (5%), pleuritic chest pain (33%), and groin haematoma (3%) (23).

### Prostate

Trans-perineal fiducial implantation was reliable and very well tolerated in our study, with complication rates close to those reported in the literature [12]. One advantage of the trans-perineal route is that it avoids perforation of the rectum, reducing the potential for infections [13, 14]. In this study, no events related to urinary infection were reported.

For intra-prostatic markers, the initial use of non-joined seeds was responsible for a decrease in the number of tracked fiducials during treatment, as the fiducials were placed too closely (less than 2 cm) and only one of the two implanted fiducials could actually be detected and tracked. Later on, by implanting joined seeds, we were able to correct this problem and increase the number of fiducials tracked during treatment sessions.

### Liver

The cube-shaped implantation pattern is a technique that uses limited cutaneous and adrenal entry ports: only one puncture was required for inserting a set of 2 markers. Thus, the very low Grade 2 complication rate (1%), lower than those reported elsewhere [15], can be partly explained by the use of joined markers; this reduced the number of needles, and therefore the number of entries and puncture-related complications. This technique, therefore, is easier to perform, less subject to complications and marker migration, and is shorter compared to other procedures [2]. In addition, the cube-shaped marker implantation pattern centred around the tumor seems to be best suited for marker recognition and tracking on kV imagery devices. In this study, marker implantation in the liver was feasible and produced a low level of toxicity. However, this was the location where the technical success rate was the lowest. On average, 25% of the implanted fiducials were not tracked since the liver is a mobile, physiologically deformable organ, which changes the geometrical relationship among the fiducials. Therefore, ideally, four fiducials should be placed around the target, in the three planes, to ensure that three fiducials are tracked during the treatment. To our knowledge, no study has reported the number of fiducials actually tracked during stereotactic radiation therapy treatment of the liver. This observation is important, since it objectively justifies the requirement of placing at least 4 markers in order to effectively track a liver target.

## Conclusion

Optimal fiducial marker implantation is essential for a successful SBRT treatment when fiducial tracking is required. Fiducial marker implantation and tracking is a feasible, well-tolerated and effective technique in SBRT treatment for extracranial tumors. Two and a half years after installing the CyberKnife®, over 3000 patients have received SBRT treatment, 616 using fiducial marker implantation and tracking. This new line of activity has allowed us to develop cooperation ties between the radiology and radiotherapy teams, and to discuss fiducial implantation upstream in order to select the best treatment strategies.

## Data Availability

The datasets used and/or analysed during the current study are available from the corresponding author on reasonable request.

## Abbreviations

CBCT: Cone Beam Computed Tomography
CTCAE: Common Terminology Criteria for Adverse Events
SBRT: Stereotactic Body Radiation Therapy
